# The Role of Nonlinear Pedagogy in Supporting the Design of Modified Games in Junior Sports

**DOI:** 10.3389/fpsyg.2021.744814

**Published:** 2021-10-29

**Authors:** Jia Yi Chow, John Komar, Ludovic Seifert

**Affiliations:** ^1^Physical Education and Sports Science, National Institute of Education, Nanyang Technological University, Singapore, Singapore; ^2^CETAPS – EA 3832, Faculty of Sport Sciences, University of Rouen Normandy, Rouen, France

**Keywords:** nonlinear pedagogy, modified games, junior sports, task modification, transfer of learning

## Abstract

Nonlinear Pedagogy has been advocated as an approach that views acquisition of movement skills with a strong emphasis on exploratory behaviors and the development of individualized movement skills. Underpinned by Ecological Dynamics, Nonlinear Pedagogy provides key ideas on design principles to support a teaching and learning approach that accounts for dynamic interactions among constraints in the evolution of movement behaviors. In the context of junior sports, the manipulation of task constraints is central to how games can be re-designed for children to play that are age and body appropriate so that the games can still capture the key elements of representativeness as compared to the adult form of the game. Importantly, these games offer suitable affordances that promote sensible play that could be transferable to other contexts. In this paper, we provide an in-depth discussion on how Nonlinear Pedagogy is relevant in supporting the design and development of modified games in the context of junior sports. Practical implications are also provided to share how games can be modified for meaningful play to emerge.

## Introduction: Practitioners as Designers of Practice

Skill adaptation begins at a young age, and the development of physical literacy is paramount in providing a sound foundation for our children to continue to engage in meaningful physical activity later in life (Rudd et al., 2021). Children can develop physical literacy through play and games from early childhood as they become more attuned to movement possibilities offered by these play opportunities (Rudd et al., 2021). It is probably common for parents and pre-school teachers to observe how the “little ones” are able to demonstrate significant changes in foundational movement skills over the pre-school years. Such skill development and learning of movement skills can continue over the life course as individuals regularly engage in physical activities through play, structured sports, or just engaging in recreational games (see Hulteen et al., 2018). Quantity of “play” time is an important factor in supporting the acquisition of physical literacy (Rudd et al., 2021). But, the quality of “play” should also feature prominently in making a difference to how children adapt and acquire movement skills.

From a practitioner point of view, the incorporation and design of effective practices would be crucial in providing the platforms and opportunities for our children to be exposed to a myriad of possibilities in developing physical literacy. Imagine the role that practitioners play in designing activities that could suitably invite children to explore different movement behaviors in contexts where they can transfer across different performance situations (see Chow et al., 2020). For example, challenging children to jump over a bar could be akin to how children may need to maneuver over an obstacle as they hike over a fallen branch in the woods. The practitioner, be it a Physical Education teacher or a coach, could be seen as a designer of practice. But what is the practitioner designing? How can the practitioner design activities effectively? What can be modified in these activities to promote more effective skill adaptation? With reference to games played at the junior levels (i.e., below 18years of age), there can be many possibilities of modifying the games such that these games can be more accessible to the children and young adults. The key is indeed in the *modification* of these games. From a Constraints-led Approach (CLA) where goal-directed behavior emerges from the interaction of key task, performer, and environmental constraints (see Renshaw and Chow, 2019; Button et al., 2020), it has been discussed at length that task constraints (e.g., task goals, rules, and equipment) can be readily manipulated by practitioners to encourage learners to search, explore, and exploit different movement behaviors that could be more functional under various contexts. However, manipulation of task constraints alone is not the answer and the emergence of movement behaviors is not unilaterally dependent on the infusion of task constraints in the learning environment. Importantly, it is the interaction between the learner, the task, and the environment that ultimately shapes the kind of movement behaviors that would be observed. Renshaw and Chow (2019) in their position paper captured the above concept nicely in highlighting the important learner-environment mutuality and reciprocity that is inherent in learning and performance. The emergence of movement behaviors is contingent on the interaction of performer, environment, and task constraints (Button et al., 2020). Underpinned by the theoretical framework of Ecological Dynamics (Araújo and Davids, 2011) which emphasizes the embedded role for physical, cognition, emotions, and perceptual skills in skill acquisition (Rudd et al., 2021), it is crucial to understand how skill development and adaptation in junior sports can be impacted by such interactive processes that are omnipresent. Critically, it is important to leverage on effective pedagogical approaches to identify relevant design principles to support our children’s involvement in junior sports. Nonlinear Pedagogy, underpinned by Ecological Dynamics, can account for nonlinearity seen in learning and provides ideas on design principles to support practitioners (Chow, 2013).

The purpose of this paper was to discuss in greater detail the relevance of Nonlinear Pedagogy in scaffolding the design of modified games in junior sports. We will also provide examples on how games can be designed effectively and explore the implications on children’s learning experiences through such modified games. Below, we begin by discussing the relevance of adopting an ecological dynamics perspective to understanding sports development.

## An Ecological Dynamics Perspective to Understanding Sports Development

Ecological dynamics has provided a coherent, biophysical scientific rationale for explaining how coordination emerges in the central nervous system and in groups of organisms (relevant to how players in team sports interact with one another), as many system degrees of freedom are continuously re-organized under constraints for achieving specific movement goals (see Kelso, 1995). The emphasis is on specifying an individualized approach to learning, where the focus on skill acquisition is related to how each learner is able to satisfy the interacting constraints that are present in specific learning situations (Chow et al., 2020).

One key aspect that demands further unpacking relates to how the emergence of human movement behaviors cannot be devoid of the inherent connection with the surrounding environment. As briefly mentioned in the introduction section, understanding the essence of learner-environment mutuality and reciprocity is key to helping academics and practitioners sense make how development of movement skills is dynamic in nature. It argues against the whole idea of the existence of a common movement pattern that is expected across all individual learners in a performance and learning context. For example, in a throw and catch activity, it would be unrealistic to expect all the children in the group to execute the overhand throw with exactly the same movement form (although we can tell that it probably looks like an overhand throw). The individual learners would differ in some ways with reference to their individual physiological structure, strength, flexibility etc. that would impact the specific way that the throw would be executed. Even for the same individual, there would be inherent differences between throwing trials when the task goal changes (e.g., throwing for accuracy vs. throwing for distance) or even when the task goal remains constant (it is impossible to use the same muscles for joint movement in exactly the same way!). In the subsequent sections of this paper, we would share specific empirical evidence to substantiate the concepts and examples surfaced here.

What would this imply for our own understanding of sports development? In any performance and learning contexts, the affordance landscape is rich and invites the learner to act (Rudd et al., 2021). Affordances (an invitation to act) will differ for different individuals across the same and different contexts. As practitioners design an assortment of practices, junior athletes can be presented with different learning and performance contexts where a myriad of affordances could be made available to them. Withagen et al. (2017) proposed that affordances can be deemed as a way to solicit invitations for action that can be discovered, explored, and exploited *via* engagements and interactions. However, the learner’s existing intrinsic dynamics (i.e., initial behavioral repertoire) may not allow the learner to fully exploit the movement possibilities present in the performance context. For example, in a climbing study by Seifert et al. (2017), it was reported that climbers were able to perceive opportunities for actions rather than just focused on specific physical properties of the climbing environment (e.g., absolute distance between holds). The invitation to act was based on what the performer can do (existing movement capabilities) with reference to the prevailing environmental conditions to try to achieve the task goal of climbing up a route. Functional movement behaviors emerge when the existing learner’s intrinsic dynamics meets the task and environmental dynamics (Chow et al., 2016). The learner’s intrinsic dynamics will continually develop and evolve over time through the processes of exploration and exploitation *via* practice, injuries, illnesses, or even when the social-cultural constraints alter. Even with reference to how an individual’s intention is shaped, Rudd et al. (2021) highlighted that the environment (inclusive of both culture and physical properties) can play a critical role in channeling decision-making on the part of the learner. The only constant is the role that learner-environment mutuality and reciprocity enacts to support skill adaptation for learners’ ability to act and move (refer to the earlier example of climbing and decision-making).

Thus, the way to effective development of skills is not undergirded by a prescribed pathway set up by the coach or a teacher. The constant interaction between the learner, the task, and the environment would indicate that this journey is a dynamic one that is constantly evolving. A recent paper by Woods et al. (2020) discussed the idea of “wayfinding,” underpinned by Ecological Dynamics, in the search and development of movement skills. It is seen as a process of embarking upon a purposeful, intentional, and self-regulated journey for exploring and moving from one region in a landscape to another. In their paper, Woods et al. (2020) described how sports practitioners are seen as “landscape designers” who can support learners to find their own way in learning movement skills by perceiving and navigating through emergent performance-related problems. This would indicate that the learner is not a passive actor in the journey of acquiring and adapting skills. Athletes and, in the context of this paper, junior athletes would learn through involvement in practices and performance environments that challenge them to be problem solvers in a self-regulated manner. What do these athletes learn from a “wayfinding” analogy? Woods and colleagues argue that through wayfinding, learners can deepen their *knowledge of the environment* (also see Sullivan et al., 2021 discussion on this) by being exposed to a continuum of affordances in the environment as the wayfinding process is one that is characterized by embodiment and embedment (i.e., not decoupling the emergence of movement behavior between the individual and the environment).

Affordances are specific to the performance context when specific transfer is the objective such that key information is relevant to highly representative performance environments (Chow et al., 2020; Woods et al., 2020). For example, practicing for a specific game play under performance context would be indicative of such a transfer. In contrast, at the other generalized end of the continuum where transfer of learning is broader, the affordances are more diverse and not matched to a particular context. The opportunity is for the young learners to acquire a range of movements that could be transferred to other similar movement contexts. Importantly, it is also about “learning to learn” (Hacques et al., 2021). Individuals learn to make decision, learn to explore, and learn to adapt, and all these can take place over a longer time scale (Hacques et al., 2021). Perhaps, many of the foundational movement skills (e.g., throwing, catching, running) practiced in the absence of a game context (or situations where the skill is to be performed) would constitute such a scenario. The invitation to act would be less specific to the particular performance context and can be utilized across more diverse situations.

The crux to understanding the role of affordances in the development of junior sports could lie in the pursuit of enrichment of learners’ affordances. Effective skill acquisition or adaptation among young athletes is hugely dependent on how extensive athletes search, explore, and exploit their individual perceptual-motor workspace (i.e., a conceptual workspace where movement solutions reside for the learner). We want these young athletes to be provided with opportunities to achieve adaptability (i.e., flexibility and stability) in the way they use their repertoire of movement skills in performance contexts (Seifert et al., 2016). Child-led play activities could be effective and relevant activities that can lead to greater exploration of movement repertoire. The role of backyard games and less structured play could in fact be beneficial in encouraging skills development among young athletes (Machado et al., 2019; Renshaw and Roberts, 2021). These games and play activities harness the natural setting and can provide more representative game contexts for which the relevant skills are performed (Renshaw and Roberts, 2021). Key perceptual information is present in many of these games to encourage a reciprocal development of functional actions that young players will find useful. Compared to more structured practice designs that may lack a crucial element of representativeness (especially those structured practices that emphasizes drills in the absence of a game context), these child-led play activities can be effective in helping the young athletes to transfer the learning from these unstructured games to actual games or performance situations.

Notably, opportunities for skill acquisition can come in various contexts. Rudd et al. (2021) nicely captured the discussion on the role that multi-sports and donor sports play in supporting skill acquisition. Using the Athletic Skills Model (ASM; Wormhoudt et al., 2018) as the platform for exemplifying how rich and varied experiences in the form of multi-sport could work, the ASM purports how perception and action are categorized into seven distinct abilities that are relevant for developing skill and expertise for athletes. Young children should be exposed to playing different sports (multi-sports) and be engaged in various physical activities to promote the acquisition of different movement behaviors that may not necessarily have a direct link to some intended target sport that the athlete have the intention to pursue eventually. For example, a young athlete could be developing a larger repertoire of movement behaviors that could be transferable across different sports by engaging in different sports (e.g., invasion games, such as rugby and basketball; net-barrier games, such as badminton and volleyball; striking games, such as baseball or cricket). These games challenge the young athlete to adopt and adapt their prevailing intrinsic dynamics to different task dynamics expected from the different games (i.e., engaging in multi-sports). Such experiences are invaluable in supporting the acquisition of movement behaviors that could enrich their existing movement repertoire for future development in some selected target games. On the other hand, donor sports are sports that have some similarities to an intended target game. One good example would be how Futsal can be seen as a donor sport for the full 11-a-side game (Travassos et al., 2017). It is not surprising that many elite football players started off playing futsal when they were younger. There is substantially greater shared affordance landscape between the donor sport and the targeted sport. Early involvement in donor sports (e.g., futsal in this example) would support later participation in the target sport (i.e., 11-a-side football).

There also lies in the role that modification of the full-adult version of these games in supporting development of junior athletes. Clearly, when junior games are played on adult pitches and courts using adult equipment, the way the game looks and feels is very different to those played by adults. Consequently, the skills and tactics adopted in attempts to be successful are influenced by the size and strength of the players, over and above their skill (Chow et al., 2020). By altering relevant task constraints like pitch sizes, net height, number of players, the modified version of these games can be made more accessible to the junior athletes. Body-scaling of playing area, targets (e.g., basket height in basketball), and implements (e.g., smaller rackets) can support the acquisition of affordances that are relevant to the athletes’ individual dynamics more effectively. The athletes’ involvement in these modified games would also have implications on how practices could be designed appropriately to encourage the emergence of functional movement behaviors.

The point about designing representative learning designs is not incoherent with the ideas about the role of donor sports or multi-sports. The key point about representative practice tasks is to provide opportunities for the learner to become more attuned to the information sources present in the performance environment and the relevant functional movement behaviors to achieve the necessary task goal (Withagen et al., 2017). With donor sports, the task dynamics would have significant similarities to the target sport, and as such, the affordances acquired would be relevant as transfer of learning can be enhanced. On the other hand, with multi-sports, the learner would be exposed to a greater range of movement possibilities through the attunement to various informational sources present across different sports contexts. For such involvement in multi-sports, it can potentially expand the repertoire of movement solutions available to the learner where greater adaptable movements (maybe even atypical ones!) can be effective in the target sports subsequently. This is where innovative, spontaneous, and individualized movement solutions become a valuable asset to the individual who has been exposed to a wide variety of sports.

More recently, a study by Barth and Güllich (2021) examining elite track and field athletes suggests that childhood/adolescent coach-led multi-sport practice was a critical factor in determining adult practice efficiency and performance improvement. In contrast, it was reported that peer-led engagement in any sport did not have significant effect on adult practice efficiency. Barth and Güllich (2021) also suggested that long-term sustainability of athletes’ development in athletics can be facilitated by childhood/adolescent multi-sport coach-led practice. Interestingly, Güllich et al. (2020) further indicated that earlier practice experiences in other sports had a lagged effect in supporting and facilitating skill learning in soccer-specific practice. Thus, the benefit of participating in multi-sports may not be seen in the shorter term. One important point here is how the practices described in Barth and Güllich (2021) are seen as coach-led vs. peer-led (or child-led). The specific micro-structure of these practices (i.e., design of practice in each session) may require further examination to help us understand the impact of such a comparison. For example, are these practices structured or unstructured? Why are there possible differences between coach-led activities and child-led activities (as mentioned earlier in this section)? What exactly is the role of the coach or teacher in these practices? The above questions probably require more extensive empirical investigations in the near future.

Thus, it can be seen that designing practices is not an easy task and a key question needs to be addressed: How can practitioners design effective practices to support junior sports development? Below, Nonlinear Pedagogy is discussed to highlight its relevance to understanding how modifications to learning contexts can promote skill development in junior sports.

## Ideas From Nonlinear Pedagogy

### What Is Nonlinear Pedagogy?

Nonlinear Pedagogy captures key design principles underpin by Ecological Dynamics to support practitioners in the design of practices with an emphasis on encouraging exploratory behaviors to develop individualized movement behaviors. Numerous papers have been written about these design principles (e.g., Chow, 2013; Chow et al., 2016; Button et al., 2020; Rudd et al., 2021). Briefly, the key design principles pertain to establishing representativeness in practice, a focus on task simplification, awareness on the impact of informational constraints, the functional role of practice variability, and constraints manipulation.

Representativeness refers to how practitioners could consider designing practices that mimic how the movement skills could be performed in actual game contexts where the relevant perceptual information would be present to provide the affordances that could lead to effective outcomes. Practicing shooting in basketball in the absence of a defender could be useful as a form of general transfer in learning but would be void of the critical perceptual information that could guide more functional movement adaptations that would be required when the junior player comes up against opponents to attempt a shot at the basket. The general transfer focus is typically seen in pedagogical approaches that emphasizes repetition and on a prescribed movement form. There is a tendency to incorporate high volume of practice trials to develop a consistent performance outcome supported by an expected movement form requirement (i.e., typically lots of drills and repetition in the absence of a simulated game environment). Such approaches have been termed in some empirical work as Linear Pedagogy (e.g., Lee et al., 2014; Roberts et al., 2020). Empirical findings on the efficacy of Linear Pedagogy have indicated that there could be deficit in retention and opportunities for exploratory behaviors as seen for example in Lee et al. (2014) for the learning of modified tennis. It was found that learners had difficulty retaining the expected (and taught) movement form for the forehand ground stroke even though there was an emphasis on the repetition of the movement form, and they exhibited fewer movement solutions. In contrast, Nonlinear Pedagogy advocates practice contexts that incorporates situations that challenges the learner to “replicate” the movement skill in different and dynamic contexts since many of these more representative “practices” will never challenge the learner in exactly the same way (i.e., the idea of repetition without repetition).

Task simplification pertains to how practitioners could consider the importance of making performance of the movement skill easier to accomplish while keeping the perception-action coupling of the movement tight. This is with reference to how a movement behavior can be simplified such that the spatial and temporal elements of the movement are maintained. This is in contrast to the idea of task decomposition where the practice of the movement is broken up into its individual parts before attempting to incorporate all parts together eventually. In Nonlinear Pedagogy, task simplification is encouraged as practitioners would want to attempt to simplify the movement to allow for success without making the practice of the movement unrepresentative of how it would actually be performed under actual performance contexts (Chow et al., 2016). For example, in junior sports, fewer players are present in a modified game or the rackets/balls used are body-scaled or modified to reduce the task difficulty. This is one form of task simplification that would be commonly found in many modified games pegged at the level of younger children playing the games.

The use of informational constraints is another key consideration that would be relevant in junior sports. Typically, there is a tendency for practitioners to use verbal instructions that emphasize form of the movement (e.g., bend your knees as you prepare to shoot in basketball, rotate your torso as you prepare to hit the ball in tennis), which unfortunately can result in greater conscious control of the movement (and this is probably seen a lot in Linear Pedagogy-type scenario). On the other hand, informational constraints that focus on the movement outcome or effect can be beneficial (e.g., trajectory of the shuttlecock in badminton and analogy of moving like a crab for sideways movement in developing foundational movement skills). Children probably have the tendency to just want to play the game and try to “win” by achieving a certain outcome without “worrying” too much about how they may achieve it. Such informational constraints could indeed be effective even of activities that could be perceived as being more movement form oriented like in dance or gymnastics. The use of analogy to describe the movement may provide less emphasis on the specific biomechanics of the action itself and thus allow for greater exploration and individualization of movement adaption (Rudd et al., 2021). From the earlier example, Lee et al. (2014) highlighted the usefulness of using such analogy in modified tennis that emphasizes movement outcome (e.g., ball flight like a rainbow and moving the racket up the slope of a mountain) which led to diverse movement solutions not predicated on a specific movement form which was just as effective as prescriptive instructions on the movement itself.

The functional role of practice variability has been espoused quite extensively, and it supports exploratory behaviors (Button et al., 2020). Variability in practice can be promoted *via* many different means through clever manipulation of task constraints. For example, the use of different size objects to be stuck or thrown, variation of rules, or an array of obstacles to maneuver. The key challenge is, however, on the extent of variability that could be infused in the practices to encourage such exploratory behaviors. The premise is probably one that needs to consider the performer constraints carefully and how it may potentially interact with the task and the environment so that an eventual movement behavior can emerge. One would design the inclusion of such variability in practice to encourage a range of movement behaviors that are not repeated in the same way in terms of movement form but rather focus on engaging the learners to attain the task goal through a demonstration of these different movement forms (Chow et al., 2016). In our view, that would be the aim of how practice variability should be incorporated and designed into the practices. It is the presence of such movement variability that learning would probably emerge more significantly. This would also be concomitant to how movement skills are relevant in dynamic performance settings (e.g., even in gymnastics where the actual execution of the vault cannot be made in exactly the same way). However, in Linear Pedagogy, the aim is to attempt to have our learners repeat the movement with minimal variability pegged to an expected movement form. This is in contrast to Nonlinear Pedagogy where multiple pathways to success are encouraged (as discussed to a significant extent earlier in the section).

The final design principle on constraints manipulation would seem to be the key pillar for all the design principles as it does underpin the enactment of the other principles. With constraints manipulation, practitioners can put in place more representative practices, simplify the task, tweak informational constraints, and of course infuse higher or lower variability in practice. A point to note here: Representativeness should pertain to the needs of the individual and the desired learning outcome. As discussed earlier in the paper with regard to representativeness and donor/multi-sports, transfer of learning should be a consideration. Thus, the art of constraints manipulation is one that is critical in the repertoire of any practitioner to attempt to deliver effective pedagogical practices within the framework of Nonlinear Pedagogy. Surely even for Linear Pedagogy, constraints (primarily task constraints) are also manipulated and used to support practices. But the key difference would be the relevant use or scaling of these task constraints to *guide exploration, search, and exploitation of individualized movement solutions in representative learning environment*. This process, that should be omnipresent in a Nonlinear Pedagogical approach, is key to how it is different to a more typical Linear Pedagogy approach.

In the following section, we discuss some of the more relevant studies in the current body of knowledge in demonstrating how features of Nonlinear Pedagogy can enhance the design of modified games in junior sports to impact movement behaviors.

## Empirical Evidence of How Nonlinear Pedagogy Can be Effective in Enhancing the Design of Modified Games in Junior Sports

In a recent systematic review (25 studies involving 989 children) about scaling equipment and play in juniors, it was shown that children preferred using scaled equipment over adult equipment, were more engaged in the task, and had greater self-efficacy to execute skills (Buszard et al., 2016a). In other words, children performed skills better when the equipment and play area were scaled because they could adopt more desirable movement patterns to the required task. In fact, the scaling of equipment (task constraint) may provide young children, who often lack the strength required to use adult equipment proficiently (organismic constraint), the opportunity to perform the necessary skills. Notably, the learners can find the optimal movement solution when playing in a match, particularly when external conditions, such as weather, are less favorable (environmental constraint). In addition, in a tennis task (e.g., forehand stroke to hit the ball to a target located 10m away), Buszard et al. (2020a) showed that scaled equipment afforded the emergence of a functional coupling between upper arm and forearm movement variability which helped regulate the distance between the shoulder and the racket. Conversely, when full-sized equipment was used, a lack of coupling was observed between upper arm and forearm movement, leading the authors to conclude that scaled equipment promoted functional movement variability, whereas full-sized equipment resulted in the freezing of mechanical degrees of freedom. More importantly, Buszard et al. (2016a) found that scaling equipment and play area in children did not only have an influence on performance and skill acquisition, but also on psychological factors and biomechanical factors. With regard to psychological factors, children playing tennis with smaller rackets (Buszard et al., 2016b), low compression balls (Farrow and Reid, 2010) on smaller courts, and lower nets (Timmerman et al., 2015; Limpens et al., 2018) reported more engagement during practice sessions compared with children playing with standard tennis balls on a full-size court. In fact, the scaled condition created an environment that increased the number of hitting opportunities, which consequently enhanced engagement in the task and match play. For instance, lowering the tennis net height to 0.65m and 0.52m led to children adopting a more attacking style of play, as evidenced by a greater percentage of successful first serves and by an increase in the number of winning rallies without an increase in errors (Limpens et al., 2018). Similar outcomes occurred in basketball as children preferred using a junior ball (instead of an adult-sized ball), a lighter ball, and a lower basket (Chase et al., 1994). Interestingly, Buszard et al. (2016a) observed that shooting performance was significantly better when children used the ball of their preference, which was typically a ball smaller than the adult-sized ball.

Scaling equipment and play area also have an influence on skill performance and acquisition because it facilitates the coupling of perception-action process and the exploration of functional movement solution, which is considered essential for coordinated movement patterns (Buszard et al., 2020a). For instance, in tennis, low compression balls bounce lower than standard tennis balls, allowing children to hit the ball in an optimal location relative to their height (i.e., waist height; Kachel et al., 2015). Furthermore, children generate greater ball velocity while maintaining hitting accuracy when using low compression balls, which indicates that children hit the softer ball with greater power and without the fear of the ball travelling too far (Larson and Guggenheimer, 2013). The “adult” practice conditions reduced the number of hitting opportunities, which effectively reduced chances for practice repetition and consequently learning. Furthermore, the combination of decreased hitting opportunities and a more difficult practice environment when practicing in “adult” practice condition resulted in fewer successful forehands and backhands relative to the scaled conditions. Mini-tennis condition of practice was found to increase the rallies length and the percentage of forehands (Fitzpatrick et al., 2017). Similar effects of scaling equipment (e.g., smaller and lighter ball, lower net) and playing area have been found in basketball, volleyball, cricket, football, and badminton, as children performed better skills, had more opportunities to practice skills (e.g., dribbling), and play matches that further look like adult form of the game (Hadlow et al., 2017; Dancy and Murphy, 2020; Ortega-Toro et al., 2020; Harwood et al., 2021).

After a 4- to 6-week intervention period, children also tend to acquire skills faster when the practice condition was scaled. However, few longitudinal studies investigated the effect of scaling equipment and playing area (e.g., Farrow and Reid, 2010; Lee et al., 2014). In particular, after a 5-week intervention, beginner tennis players further enjoyed learning and experienced greater hitting opportunities of backhand and forehand when ball and court size were modified in comparison to adult standard ball and court. After a 4-week intervention, children involved in a Nonlinear Pedagogy (i.e., manipulation of task constraints including equipment and rules) to learn the tennis forehand exhibited more movement solutions (referring to degeneracy of perception-action system) in organizing limbs and racquet (in particular right and left elbow flexion/extension, right and left shoulder internal/external rotation, and racquet rotation angle) than children who received a Linear Pedagogy (i.e., prescriptive, repetitive drills; Lee et al., 2014).

Interestingly, when biomechanical factors were examined, like scaling equipment size to children anatomical measures, low or even nonsignificant correlation were found on performance (Chase et al., 1994; Gagen et al., 2005; Buszard et al., 2014). Limpens et al. (2018) argued that the net height in tennis should be approximately 50% of children’s height given that the full-size net height is about 50% of the professional tennis player’s height. This logic of scaling implies that the aim is to maintain a similar ratio between physical size (anatomical measures) and task constraints (equipment and environments) in children and adults. However, this means that equipment and environments follow a linear scaling process toward adult anatomical measures (Buszard et al., 2020b). This assumption does not match with recent findings highlighting nonlinearities in the dynamics of learning. This assumption does not match with recent findings highlighting nonlinearities in the dynamics of learning. Therefore, further research is required to understand how scaling equipment to children’s anatomical measures could have an impact on skill acquisition. Indeed, variable practice (like equipment size) is one ingredient of Nonlinear Pedagogy that was advocated to develop adaptive learning behavior and skill transfer (Chow, 2013; Lee et al., 2014). In fact, Nonlinear Pedagogy goes beyond scaling equipment and play area to children possibilities and anatomical measures; therefore, this scaling should be accompanied by other principles promoted by NLP, such as (i) saving the representativeness of the learning design, (ii) favoring perception-action coupling by promoting match play, (iii) using variable practice, and (iv) manipulating attentional focus. As an example, van den Tillaar and Marques (2013) compared the effect of specific and variable practice in overhead throwing task by varying the ball weight in 41 children of 7.7years. They compared throwing speed and distance of children throwing a soccer ball or 1kg balls with children using variable practice with 0.35, 0.45, 0.5, and 1kg balls. After a 6-week period of practice, both specific and variable practice of throwing in children lead to increases in performance. However, it seems that the increased workload (practice) is a more important factor than the type of practice (specific or variable) in enhancing performance in children. While previous studies did not show evidence of variable practice in children movement skill (Moore et al., 1981; Clifton, 1985), recent review papers (Ranganathan and Newell, 2013; Pacheco et al., 2019; Ranganathan et al., 2020) emphasized the interest of variable practice to develop flexible movement skill and effective searching strategies.

From the above list of work that have been discussed, it can be seen that there are on the whole, many benefits to modifying the games or performance context with reference to junior sports. As clearly seen, body-scaling equipment can help junior athletes develop more functional movement behaviors. Adjusting the use of projectiles (e.g., characteristics of balls used) can also simplify the task for the learners without sacrificing the critical perception-action couplings present in the games. This lends reference to the pertinent role of effective manipulation of task constraints to make the practice representative for the junior athletes so that functional affordances can be acquired when the learners are engaged in such practices. As can be seen, the key design principles espoused in Nonlinear Pedagogy are relevant in supporting practitioners in creating learning environments that encourage exploration of individualized movement solutions. By experiencing success, the junior athletes can also be more motivated to continuously engage in sports. This has important implication in relation to talent development (as mentioned earlier) and can potentially provide stronger foundation for life-long engagement in physical activity.

In the following section, we explore more about suggestions on how Nonlinear Pedagogy can be applied in junior sports.

## Applications of Nonlinear Pedagogy in Junior Sports

### Small-Sided and Conditioned Games

In practice, modified games became highly popular in the last few decades, specifically in national sports programs. Modified sport programs are offered to primary school-aged children (generally 4 to 12years) all over the world, although the upper age limit varies from program to program. Modified sports include diverse activities from Australian Football League (2014), Netball Australia (2014), Football Federation Australia (2015), Tennis Australia (2015), French Handball Federation (2019) (Handball), and French Rugby Federation (2021) (Rugby) among others. Specifically, modified sport programs are offered to engage children in play activities to develop fundamental motor skills and sport-specific skills for future performance (Coté et al., 2019). Essentially, the sport is modified to match the developmental capabilities of children by adapting games and activities through changes to the rules, equipment, and/or physical space to encourage inclusion and maximize participation (i.e., key manipulation to task constraints). The fundamental focus of modified sport programs is on learning and development, including developmentally appropriate competition, rather than on the competition *per se*. Modifying the game can be seen as a way to ensure representativeness of the practice. But, the purpose of training and learning modifications of the games can also serve as a powerful tool to implement the key pillars of Nonlinear Pedagogy.

In this context, small-sided and conditioned games (SSCG; commonly used modified games that take place in tight spaces, involving small numbers of players and with modified rules of the game) have been proposed to be an effective methodological tool for optimizing the tactical behavior of athletes (Davids et al., 2008; Orth et al., 2012). More than just an immediate effect on the performance or game play, SSCG is one of the strategies that can be employed from a CLA in order to improve learning (Davids et al., 2008). Indeed, the careful manipulation of constraints, as a way to guide emerging behaviors but not prescribe them, appears to be the key principle of Nonlinear Pedagogy applied to modifying games (as seen from the in-depth discussion in the earlier sections). A large body of literature focuses on modified game in football (soccer), specifically on the effect of SSCG. At all ages (youth but also adult), numerous impacts of SSCG appear in the organization of the play, physical demands of the game, individual involvement of players in the game (see Aguiar et al., 2012 for a comprehensive review on the modified games format in football and their effect). Based on the idea of constraints manipulation, SSCG can potentially be of high interest in influencing specific skill sets of players beyond just merely scaling the equipment to better fit the developmental level of the children. In other words, SSCG can modify the way the players are playing without prescribing a particular way. Orth et al. (2012) confirmed how functional interpersonal interactions between attackers and defenders in football, based on variations in key task constraints, such as relative spatial positioning between attackers, defenders, the ball, and goal during performance, can shape skill performance and decision-making behaviors (Orth et al., 2012). In that sense, if the modified game is specifically designed to those key constraints that control player’s behavior, it can become a power tool to guide learning. From this perspective, Headrick et al. (2012) established how emergent actions and decision-making of players can be modulated in modified games by manipulating specific practice task constraints. For example, the on-field location for dyadic system interactions (Headrick et al., 2012), scaling performer physical characteristics, such as height, changing initial distances between defenders or between defenders and attackers (Orth et al., 2012), manipulating instructional constraints on players (conservative or risk-taking instructions), and modifying playing rules (Passos et al., 2011). The effects of SSCG are very broad and go beyond the simple increase in immediate performance (i.e., due to equipment scaling) and can entirely reorganize the way the children play the game and organize themselves.

### Beyond Small-Sided and Conditioned Games: Relevance of Task Modification

While SSCG is probably the most popular, it is not the only way to modify games to impact players’ behaviors and learning. Recently, Oppici et al. (2018) showed that using a modified ball in football can impact the perceptual ability of players. Indeed, they showed that using a modified ball as compared to a regular ball for training in football led to improved passing performance but also specifically to changes in perceptual attunement. Those results corroborated with previous empirical work, showing that involvement in futsal practice early on could enhance later football performance and skill mastery (Travassos et al., 2017). The authors proposed that the ball or equipment in general as a task constraint can be modified to influence the learning process. Other constraints can also be modified in the game (e.g., scoring rules, number of players, and court dimensions) to positively impact learning on players. Therefore, SSCG is only one specific approach within modified games as a form of constraint manipulation, and evidence of positive effects of other modified games perspectives has been highlighted in football and several different activities.

A recent study in field hockey showed that using a modified ball led to a significant improvement in field hockey skills (Brocken et al., 2020). More specifically, the authors demonstrated that the modified equipment increased learners’ movement variability, which is proposed to be the foundation of their skill improvement. Brocken et al. (2020) showed that manipulation of the ball characteristics not only improved the current performance of the players (because it is easier to use), but also benefited learning by promoting movement execution redundancy. As mentioned in the earlier section, Limpens et al. (2018) showed how modifying net height could immediately impact movement behavior of players. Specifically, decreasing the net height significantly improved the percentage of winners. Conversely, with increased net height, the players exhibited shorter rally (i.e., less shots per rally). Interestingly, the hypothesis in this study was based on the net height in relation to a percentage of child height at the age of practice (i.e., based on the average height of 10years old), which represents an equipment modification scaled to individual constraints of the players. Such an approach to modifying the game or equipment on the basis of the players’ constraint represents a relevant way to manipulate task constraints regardless of the players’ ability. This is a novel way of manipulating task constraints that promotes task simplification, that is, provides a solution for the junior athletes to be successful in the task regardless of their current capacities (Limpens et al., 2018) but still maintains a strong and relevant coupling between perception and action (Davids et al., 2008).

### Challenges in the Use of Nonlinear Pedagogy

Nevertheless, while there are advantages in the use of Nonlinear Pedagogy, challenges would and could still surface for practitioners. Chow (2013) indicated some of these challenges pertaining to the need to know the sports/game/activity well so that appropriate manipulations of constraints can be enacted to effectively channel learners’ search behaviors. In addition, it can potentially be time-consuming to prepare the relevant activity area or equipment and this is especially in the context of a school setting where the teacher may need to transit between lessons for different groups of students. Prior and careful preparation for teaching and learning will need to be organized to facilitate the delivery of Nonlinear Pedagogy-based lessons. Practitioners may also feel uncomfortable initially when using a Nonlinear Pedagogy as there could be a sense of lost in control of how the session may progress since less prescriptive instructions are made available for the students even though autonomy of learning would then lie in the hands of the learners. There is a need to be seen in control of the learning environment, and some practitioners may potentially be uncomfortable with such a context (Chow, 2013).

## Conclusion

This paper has provided a brief discussion on what Nonlinear Pedagogy is and how it can be relevant in supporting the design of modified games for junior sports. The key design principles in Nonlinear Pedagogy are undergirded by Ecological Dynamics and focus on the role of constraints manipulation in supporting an environment that promotes skill adaptation that would be functional for junior athletes. Importantly, we see the potential of Nonlinear Pedagogy in impacting curriculum design and policy on the development of junior sports. From this paper, we have highlighted evidence from previous work that have positively supported the emergence of movement behaviors that are effective for young athletes in the modified versions of the games. We have also presented ideas on how principles of Nonlinear Pedagogy could be applicable to support practice and competition designs in national sports programs. Children are not little adults, and practitioners should be cognizant of how adjustments to task constraints can interact with the performer and the environment to effect different movement possibilities for the junior athletes. The design of practices is an art as much as it is also underpinned by science. Nonlinear Pedagogy does not advocate or prescribed a specific formula to consider developing modified games in junior sports. Rather, Nonlinear Pedagogy provides a framework for the practitioners to consider in terms of the “what,” “why,” and “how” of using the design principles as espoused in Nonlinear Pedagogy to support how junior athletes “wayfind” their own skill development journey as they transition into the domain of the full game.

## Author Contributions

JC conceptualized and wrote the paper. JK and LS assisted in the conceptualization of the paper and wrote sections of the paper. All authors contributed to the article and approved the submitted version.

## Conflict of Interest

The authors declare that the research was conducted in the absence of any commercial or financial relationships that could be construed as a potential conflict of interest.

## Publisher’s Note

All claims expressed in this article are solely those of the authors and do not necessarily represent those of their affiliated organizations, or those of the publisher, the editors and the reviewers. Any product that may be evaluated in this article, or claim that may be made by its manufacturer, is not guaranteed or endorsed by the publisher.
